# Hypha essential genes in *Candida albicans* pathogenesis of oral lichen planus: an in-vitro study

**DOI:** 10.1186/s12903-021-01975-5

**Published:** 2021-12-01

**Authors:** Hong He, Ying Wang, Yan Fan, Congcong Li, Jianxin Han

**Affiliations:** 1 Stomatology Hospital, School of Stomatology, Zhejiang University School of Medicine, Zhejiang Provincial Clinical Research Center for Oral Diseases, Key Laboratory of Oral Biomedical Research of Zhejiang Province, Cancer Center of Zhejiang University, Hangzhou, 310006 China; 2 Hangzhou Stomatology Hospital, Pinghai Road, Hangzhou, 310000 China; 3 Department of Food Science and Nutrition, School of Biosystems Engineering and Food Science, Zhejiang University, Hangzhou, 310006 China

**Keywords:** Bimorphysim switch, *Candida albicans*, Hypha/mycelium essential genes, Oral lichen planus, Pathogenesis, Etiology

## Abstract

**Background:**

Hypha essential genes (*HEGs*) of *Candida Albicans* have been emerging into scholar’s attention, little known about their functions in oral lichen planus (OLP) with an uncovered etiology. This research aimed to observe necessary genes in biphasic *C. albicans* from OLP and study their relevance in pathogenesis, so as to evaluate possible roles of morphologic switching in etiology of OLP.

**Methods:**

Samples were collected from OLP lesions of patients, mycelia were cultured and total RNA was extracted then subjected to reverse transcription-PCR and real-time PCR.

**Results:**

*HWP1* and *HGC1* were significantly expressed in hyphae phase and weakly detected in yeast phase, while there was no significant difference of *EFG1, ALS3, and ECE1* between in yeast and mycelia.

**Conclusion:**

*HGC1* and *HWP1* were confirmed to be hypha essential genes, with HGC1 for hypha morphogenesis and *HWP1* for adhesion invasion in pathogenesis of *C. albicans* in OLP. *ALS3, ECE1* and *EFG1* played minor roles in hyphae maintenance and adhesion for hyphae. These might be deemed as hints for the etiology of OLP and indicate *HGC1* and* HWP1 *to be a priority of potential drug target.

## Background

The sense of *Candida albicans* (*C. albicans*) in oral lichen planus (OLP) has acquired extensive attentions [1–3]. The course of pathogenesis of *C. albicans* usually includes three stages: adhesion, invasion and tissue lesion, in which hypha growth is the vital stage, for that yeast cells and germ tube could be swallowed by neutrophils, while the hyphae and long germ tube wouldn’t [4], with a consistence to the common knowledge that hyphae cells might inhibit chemotaxis, absorption and consuming of neutrophils, and avoid being disrupted by phagocytes. What is its role in OLP?

**Fig. 1:**
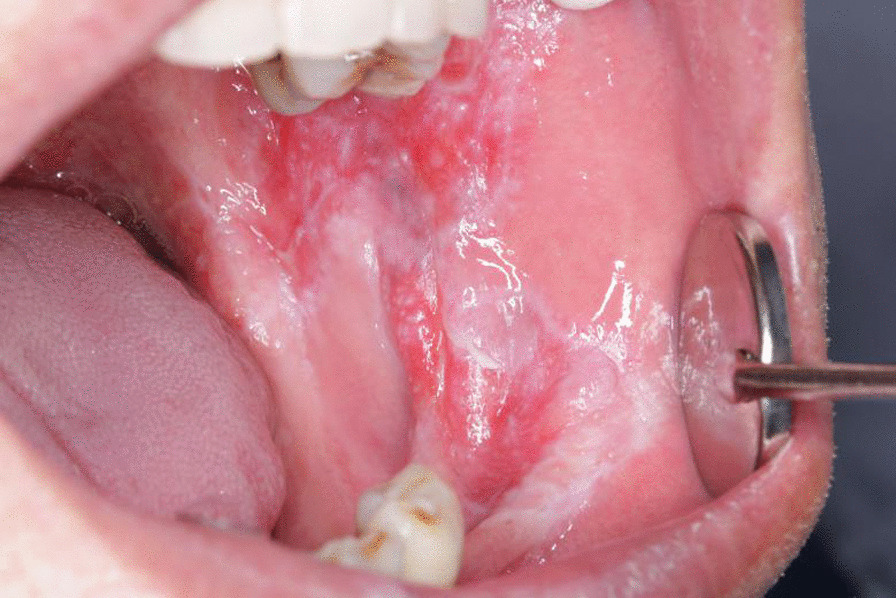
The typical erosive lesion of patients with OLP

Probable genotypic mutations of *C. albicans* in the occurrence and development of OLP (Fig. 1) were illustrated in previous researches [5, 6]. Further studies about phase transformation of *C. albicans* in OLP progression have been conducted. Change of the gene expression was uncovered to be directly related to phase switching of *C. albicans*. Specific expressions of *HGC1, HWP1, ECE1, ALS3,* and *EFG1* in yeast and hypha phase of *C. albicans* from OLP were detected after RNA isolation, function and significance of these hypha essential genes (*HEGs)* and signal molecule *EFG1* were analyzed in this study.

## Materials and methods

Enrolled subjects included 59 patients with non-erosive OLP and 41 patients with erosive OLP visited dental school of Zhejiang University during 2011.8.1- 2015.6.1. The patients were clinically diagnosed and histopathologically confirmed as OLP, with no history of smoking and alcohol abuse; moreover, they hadn’t got any visible oral lesions except OLP; besides, they were off systemic or topical anti-inflammatory or immunosuppression/immunomodulatory drugs; furthermore, they received no treatments for OLP within 3 months prior to the specimen collection, and without any relevance to systematic infections, allergies, cardiovascular diseases, immunodeficient diseases and autoimmune diseases.

The non-erosive group of 59 patients included an average age of 53.24 years old, 23 males averaged 53.84 years old and 36 females averaged 52.62 years old. The erosion group of 41 patients included an average age of 52.98 years old, 14 males averaged 48.70 years old and 27 females averaged 55.99 years old. There were no significant differences of gender or age between the two groups.

The study was carried out in accordance with the Helsinki Declaration of 1975, as revised in 2000, and was reviewed and approved by the local ethic institutional review boards (IRB) raising the ethic codes as No. 2010 (137) from the second hospital and No. 20150012 from the stomatology hospital both affiliated to the School of Medicine Zhejiang University. All the patients delivered their written consent for participation in the study.

### Equipment and reagents

Refrigerator (− 4 °C, − 20 °C) (Haier, China), Water bath (Grant, China), 4 °C Centrifuge (Hettich, China), Electric centrifuge (Poly scientific instrument co., LTD., Jiangyin city, Jiangsu province), Centrifuge (Heraeus, France), RNA/DNA Calutater (Thermo, China), Pipetting gun (Gilson, France), PCR machine (Tpersonal, Germany), real-time PCR machine (AB stepone plus, USA), Gel imaging analysis system (Cell Biosciences, Germany), Electrophoresis apparatus (Amersham Pharmacia Biotech, China).

CTAB, DEPC (Hangzhou heaven biotechnology science co., LTD), Glass beads (Hangzhou heaven biotechnology science co., LTD), Agarose (Gene Tech, Shanghai), TBD (Chinese academy of medical sciences, bioengineering institute for medical research), Chloroform, Analysis alcohol (Shanghai LingFeng biological technology co., LTD), 25:24:1 Phenol/Chloroform/Isoamyl alcohol (Bioflux, China), real-time PCR kit (Applied Biosystems, USA), 600 bp/1000 bp/4000 bp D (NA ladder Marker Bioflux, China), BikReady rTaq, 10*PCR Buffer (Bioflux, China), dNTP Mixture (Insite, China), OligdT (Invitrogen, China), 25 mM MgCl_2_ (Fermentas, China), M-Mlv reverse transcriptase, 5*Buffer, Rnasin Ribonuclease Inhibitors (Promega, USA), Gelred (Biotium, USA), SYBR real-time PCR premixture (Applied Biosystems, USA), RT-PCR primer (Synthesized by Shanghai sangon biological engineering co., LTD), real-time PCR primer (Synthesized by Shanghai sangon biological engineering co., LTD).

### Methods

Samples were collected by rubbing with sterile cotton swab in lichen planus lesions, and then transferred within 30 min to clinical microbiology laboratories of Affiliated 2^nd^ Hospital, School of Medicine, Zhejiang University.

#### Preliminary identification of the *C. albicans* strain

Twist cotton was coated on Saori Lloyd agar, cultured for 24–48 h at 37 °C until bacterial colonies appeared, and then transferred to French CHROMagar chromogenic medium. After 24 h, colonies showing emerald were initially identified as *C. albicans*. Colonies appeared dark green were suspected as *C. parapsilosis*. Suspected *parapsilosis* were rechecked after another 24–48 h culturation in SDA for the reason of similar color. If it showed emerald green, *C. albicans* was confirmed; if it remained dark green, *C. parapsilosis* was identified.

#### Further characterization, separation and purification

Typical colonies were picked and inoculated on Sabouraud Lloyd agar plates three times for separation and purification. After being cultured for 48 h in SDA, the colonies of the isolates were milky white cheese-like circular shape, formed germ tube after 4–6 h 37 °C in the rice Tween agar, grew well after being incubated for 48 h in SDA. Light microscopy showed Gram-positive structures with large cell volume, circular or oval layers by the modified Gram staining. API 20C AUX Candida identification system confirmed that the results were in accordance with what in databases provided by BioM6rieux. Finally, cinnamon peptone strains were stored in − 20 °C refrigerator and were regularly sub-cultivated.

Strains were marked as isolates a–h, among which a/c/g were collected from female non-erosive OLP patients, b/e/ f were from female erosive OLP, isolate d was from male-erosive group, and the last isolate h was a standard strain (ATCC16220) thankfully afforded by clinical microbiology laboratories of Affiliated 2nd Hospital, School of Medicine, Zhejiang University.

#### Yeast phase culture of *C. albicans*

Two mm diam. of yeast colonies were picked into cell culture flask with 5 ml YPD (121  °C, 30 min autoclaved) with an adjusted concentration of 1*10^6^/ml and were shaken in 37 °C, 200 rpm thermo shaker for 24 h. The yeast phase was cryo preserved in −20 °C.

#### Hyphal phase culture of *C. albicans*

Two mm diam. of hyphal colonies were picked to water-jacket thermostatic constant incubator with 4.5 ml Roswell Park Memorial Institute (RPMI) 1640 + 0.5 ml calf serum (56 °C, 30 min inactivation complement). The concentration was adjusted to 1*10^6^/ml for culture 7 days and were passaged 12 times (the 1st 2 days, passaged every 8 h; the medium 2 days, passaged every 12 h; the last 3 days, passaged every 24 h). Finally, the hyphal collection was up to 99%.

In addition, 5 ml RPMI 1640, 4.5 ml RPMI 1640 + 0.5 ml calf serum (no inactivation) and 4.5 ml RPMI 1640 + 0.5 ml calf serum (121 °C, 30 min inactivation) were set as controls, mycelium cell formation was observed. The number of cells forming hyphae among 100 cells was counted at 2 h, 3 h, 6 h, 8 h, 12 h, 24 h, and 7 days under high power microscope three times for an average hyphae formation rate.

#### RNA extraction

The total RNA of biphasic *Candida albicans* from eight strains of 16 samples (each 5 ml) was extracted by acid-washed glass bead method and modified hot acid phenol method. The yeast phase strains were labeled as isolates ay–hy and hyphal phase strains were labeled as isolates ah–hh. 260/280 and DNA concentrations and purities were averaged after three repeats of performance.

#### Reverse transcriptase‐polymerase chain reaction (RT-PCR)

Total RNA (2 µl) was reverse-transcribed into complementary DNA (cDNA) by incubation with 1 µl of reverse transcriptase in 20 µl of reaction buffer containing 1 µl of random primers and 10 mM dNTPs at 42 °C for 1 h. Then 2 ng of cDNA was used as the template for PCR. The PCR reaction parameters of 18s rRNA were: 35 cycles of 94 °C for 2 min, 94 °C for 30 s, 50 °C for 30 s, 72 °C for 40 s and 72 °C for 10 min; the PCR reaction parameters of *EFG1, ECE1, ALS3* were: 35 cycles of 94 °C for 2 min, 94 °C for 30 s, 52.5 °C for 30 s, 72 °C for 1 min and 72 °C for 10 min; and the reaction parameters of *HGC1, HWP1* were: 35 cycles of 94 °C for 2 min, 94 °C for 30 s, 53 °C for 30 s, 72 °C for 45 s and 72 °C for 10 min. Relative primer sequences used were listed as in Table 1.

**Table 1:** RT-PCR and real-time PCR primer

Primer	Sequence
**RT-PCR**	
18s rRNA+	5′-GGGGATCGAAGATGATCAGA
18s rRNA−	5′-CACGACGGAGTTCACAAGA
HGC1+	5′-CATTAACTCCAAAATCAATTTCACAACA
HGC1−	5′-ATCGAGTTTTAGTATA A ATAGGAGA
EFG1+	5′-ACGGAAATTACAATAACGGTATGCCC
EFG1−	5′-TTCTTTGGCAACAGTGCTAGCTGAT
ECE1+	5′-ATTCTCCAAAATTGCCTGTGCTA
ECE1−	5′-AGCTTTTCCGA A ATATTCTTCAATC
ALS3+	5′-TGTTACTCATATATTTGTCGGTTGC
ALS3−	5′-ACATGGTGTCATAAATAATCACAG
HWP1+	5′-ACTGCTCAACTTATTGCTATCGCTT
HWP1−	5′-TGTTACCAGCACCTTCAAAGTAGA
**Real-time PCR**	
18s rRNA+	5′-TCTTTCTTGATTTTGTGGGTGG
18s rRNA−	5′-TCGATA GTCCCTCTAAGAAGTG
HGC1+	5′-GGTAACACCACCAAATC
HGC1−	5′-GAAGAAACAGCACGAGA
EFG1+	5′-TTCTGCTTCGGCTCCTC
EFG1−	5′-CTGCTGTTGGTTGTAAGTTTGA
ECE1+	5′-GGCAACATTCCACAAGTA
ECE1−	5′-AGGACGCCATCAAAAA
ALS3+	5′-GTGACCACCTTACCATTCG
ALS3−	5′-ACAGTAGCAGTTTCCCCAAT
HWP1+	5′-CGGAATCTAGTGCTGTCGTCTCT
HWP1−	5′-TAGGAGCGACACTTGAGTAATTGG

#### Real-time polymerase chain reaction (Real-time PCR)

Real-time PCR was performed using real-time PCR kit (Applied Biosystems, USA). The reaction parameters were: 35 cycles of 94 °C for 5 min, 94 °C for 30 s, 50 °C for 30 s, 72 °C for 30 s and 72  °C for 10 min. Real-time PCR primer sequences were also listed as in Table 1.

After each template and gene amplification, a program was set: 95 °C for 15 s, 62 °C for 20 s, temperature 62 °C slowly increased to 95 °C for 15 s within 20 min. Continuously the fluorescent signal of sample was collected in the process of climbing to get the melting curve, and the melting curve was available through quantitative real-time PCR own analysis software.

#### Statistical analysis

A *P* value < 0.05 was set as the standard statistical significance. The statistical comparisons of the experimental data were performed by one-way ANOVA using SPSS statistical software (SPSS 25.0; SPSS Inc., Chicago, IL, USA).

## Results

### *Candida* and *C. albicans* detection results from OLP

After the phenotypic characterization from Sand type medium cultivation, Secco ma jia chromogenic cultivation, and separation, there were 5 cases of *C. albicans* and 1 case of *Candida tropicalis* from 59 cases outcomes of non-erosive OLP, and 7 cases of *C. albicans*, 1 case of *Candida krusei* and 1 case of *C. parapsilosis* from 41 cases of OLP erosive type. The *Candida* and *C. albicans* detection results were reported in Table 2. Additionally, the test results of CD3^+^ CD4^+^ T_h_ cells in patients were also recorded in Table 3.

**Table 2:** *Candida* and *C. albicans* rates in patients with erosive/non-erosive OLP

Group		*Candida* positive cases (rate)		*C. albicans* positive cases (rate)
OLP-ne (n = 59)	Male (n = 23)	2 (8.70%)	6 (10.17%)	5 (8.47%)
	Female (n = 36)	4 (10.26%)		
OLP-e (n = 41)	Male (n = 14)	3 (21.43%)	9 (21.95%)	7 (17.07%)
	Female (n = 27)	6 (22.22%)

**Table 3:** Average T_h_ cells in blood of patients with OLP

Group	Average of CD3 + CD4 + (%)	
	helper/induced T cells	Normal range
Candida negative group Candida positive group	37.40^*^ 33.27^*^	27–51 27–51

^*^ Averaged including a certain proportion of patients less than the offline

### The electrophoresis for RT-PCR products of *HEGs* and *EFG1*

**Fig. 2:**
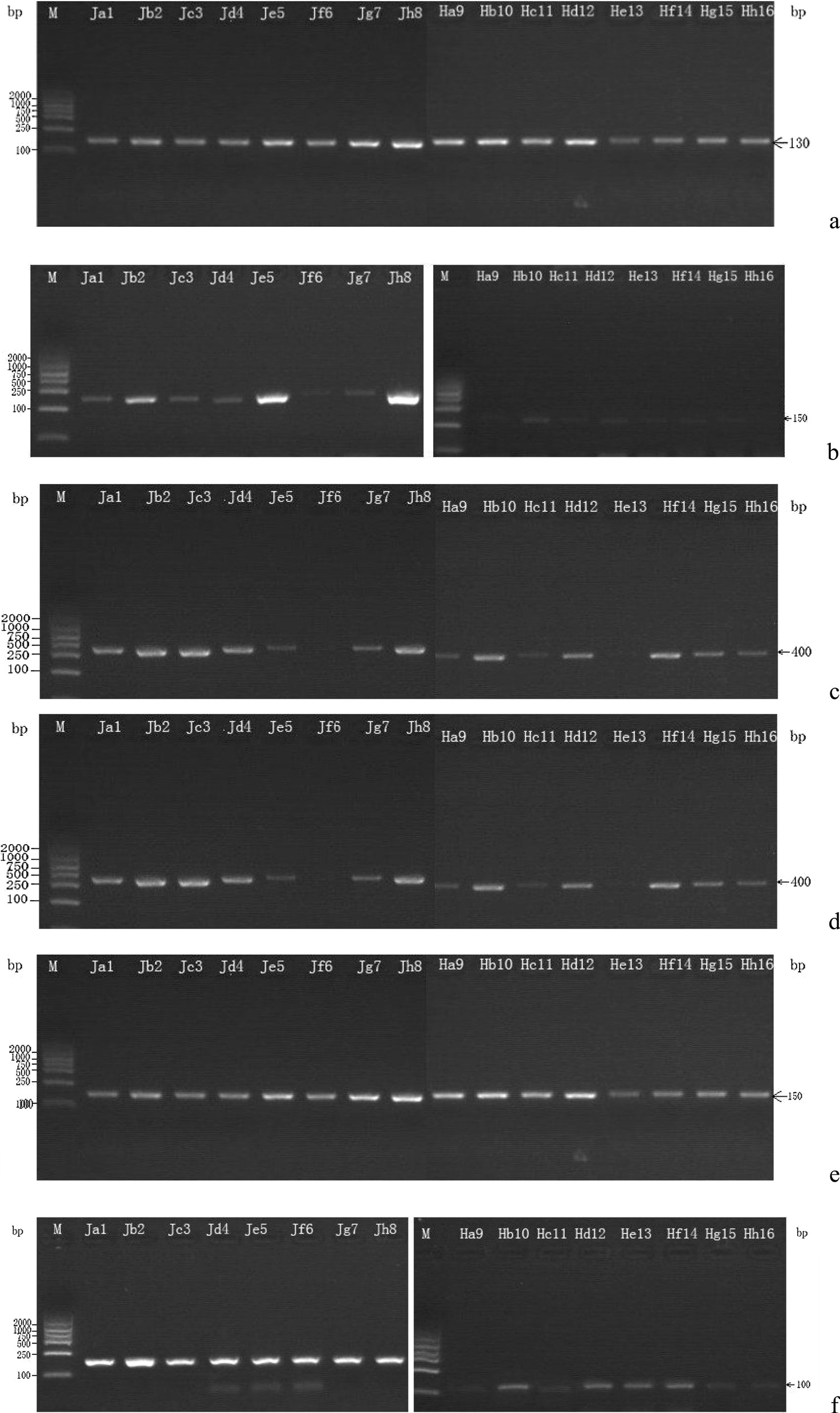
**a–f** 2% agarose gel electrophoresis of genes of biphasic cells (mycelial phase and yeast cells): from left to right in turn were 8 strains of yeast cells, 8 strains of hypha cells, and the reference 18s ribosomal RNA; from up to down successively were 18s ribosomal RNA, *EFG1, ALS3, ECE1, HGC1* and *HWP1*. Products showed the significant expression of *HWP1* and *HGC1 *mRNAs in mycelial phase cells rather than in yeast

Figure 2a–f was the electrophoresis of the expression of *HEGs*, in which *EFG1, ALS3, ECE1* were expressed in both phases; *HGC1* and *HWP1* showed no expression in yeast phase.

### The real-time PCR results of each *HEGs* and *EFG1*

**Fig. 3:**
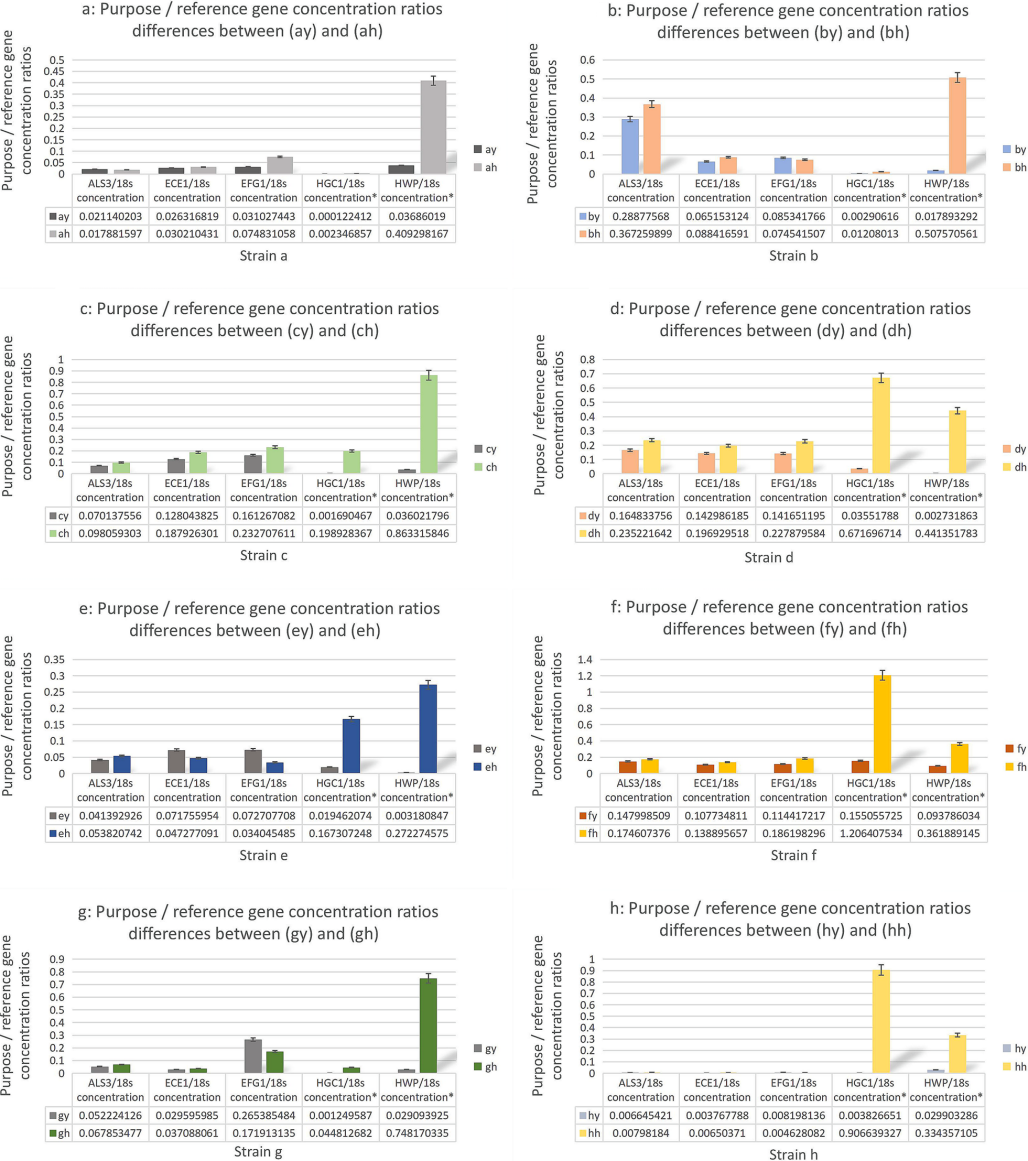
**a–h** Purpose / reference gene concentration ratios between yeasts (y) and hyphea (h) of *C.albicans* strains a-g from OLP

**Fig. 4:**
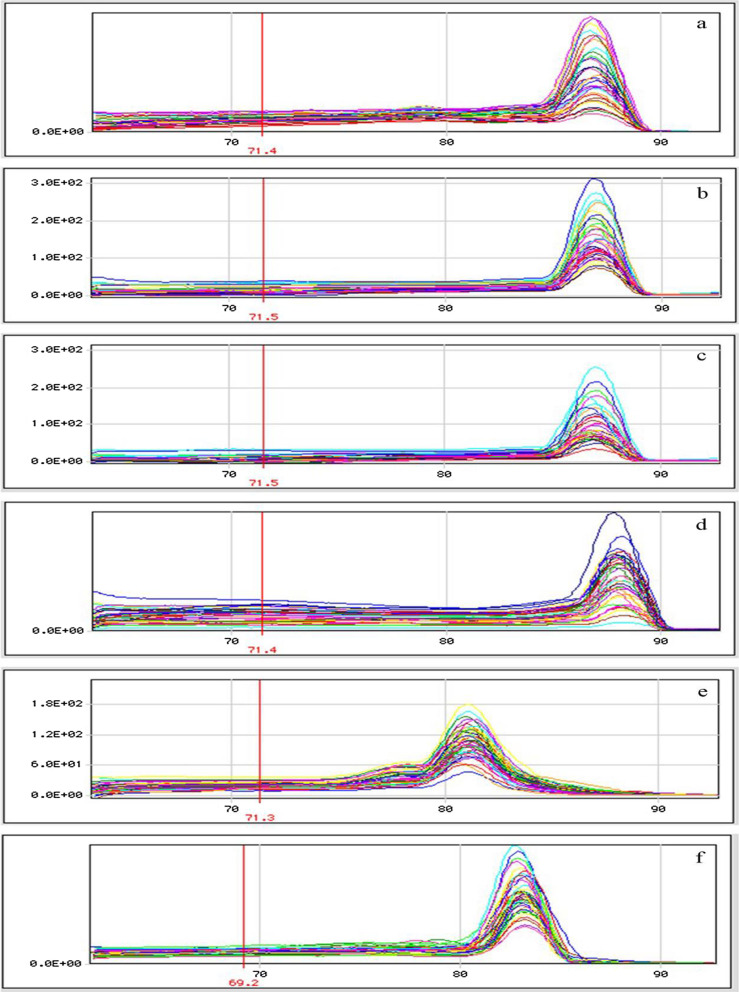
Melting curves: **a** for *ALS3*, **b** for *ECE1*, **c** for *HGC1*, **d** for *HWP1*, **e** for *EFG1* and **f** for 18s rRNA. Note: The unique peaks in melting curves present sound specificity of amplification, and the peak positions near the annealing temperature indicate effectiveness of results

The results of real-time PCR for *HEGs* (*HWP1, ECE1, ALS3, HGC1, EFG1*) were shown in Table 4, Fig. 3a–h and 4 which showed that *EFG1, ECE1, ALS3* got a ratio as *P* > 0.05 in biphasic cells comparing with the internal reference genes with no significant difference, while *HGC1* and *HWP1* got *P* < 0.05, namely there were significant differences for *HGC1* and *HWP1* between biphasic cells.

**Table 4:** Purpose / reference gene concentration ratios between yeasts (y) and hyphea (h) of *C.albicans* strains a-g from OLP

Strain	ALS3/18s concentration		ECE1/18s concentration	EFG1/18s concentration		HGC1/18s concentration	HWP1/18s concentration
ay	0.021140203		0.026316819	0.031027443	0.000122412		0.03686019
ah	0.017881597		0.030210431	0.074831058	0.002346857^*^		0.409298167^*^
by	0.28877568		0.065153124	0.085341766	0.00290616		0.017893292
bh	0.367259899		0.088416591	0.074541507	0.01208013^*^		0.507570561^*^
cy	0.070137556		0.128043825	0.161267082	0.001690467		0.036021796
ch	0.098059303	0.187926301		0.232707611	0.198928367^*^		0.863315846^*^
dy	0.164833756	0.142986185		0.141651195	0.03551788		0.002731863
dh	0.235221642	0.196929518		0.227879584	0.671696714^*^		0.441351783^*^
ey	0.041392926	0.071755954		0.072707708	0.019462074		0.003180847
eh	0.053820742	0.047277091		0.034045485	0.167307248^*^		0.272274575^*^
fy	0.147998509	0.107734811		0.114417217	0.155055725		0.093786034
fh	0.174607376	0.138895657		0.186198296	1.206407534^*^		0.361889145^*^
gy	0.052224126	0.029595985		0.265385484	0.001249587		0.029093925
gh	0.067853477	0.037088061		0.171913135	0.044812682^*^		0.748170335^*^
hy	0.006645421	0.003767788		0.008198136	0.003826651		0.029903286
hh	0.00798184	0.00650371		0.004628082	0.906639327^*^		0.334357105^*^

^*^P < 0.05

## Discussion

### Existence of *C. albicans* in OLP

Researches about *C. albicans* as an opportunistically infectious fungus started from 1940, and its relationships with OLP has also drawn attention of scholars since 1974 [1, 7]. Non obvious correlation between *C. albicans* and OLP was collected through a phenotypic characterization and in reports of Lundstrom [8] later in 1984, Lipperheide [9] in1996, and Mehdipour [10] in 2010.

On the other hand, genotype identification of clinical isolates by Jainkittivon [11] in 2007, Zeng [12] in 2009, and phenotypic characterization by Hatchuel [13] in 1990, as well as the computer selection by Li [5] in 2011 and our studies etc. have come to show that *Candida* comorbid rate of OLP was obviously higher than that of ordinary normal crowd, and certain differences of *Candida* comorbid rates have been also discovered between erosive and non-erosive OLP. These results of clinical detection rates of *C. albicans* were illustrated in Table 2.

Here reported, the *Candida* infection rates for 59 cases of non-erosive OLP group and 41 cases of erosive OLP group were 10.17% and 21.95% respectively, and the *C. albicans* infection rates were 8.47% and 17.07%, being consistent with the results of previous research [12].

### Phase switching of *C. albicans* in OLP

After colonizing in the surface of oral mucosa, *C. albicans* would adhere to the epithelium with the help of mycelium and germ tube. When host defense gets weaker, the fungi would invade the epithelium, escaping from host defense by mycelium morphology to cause opportunistic infection [14, 15]. Correspondingly, the average age of patients with OLP in this experiment was 52.23 years old, and the women patients aged 45–52 were relative to be menopausal. Literatures showed that estrogen receptor [16] (ER) levels in male and middle-aged women patients with OLP were both lower than healthy individuals, and their cortisol levels [17, 18] were higher than normal control group. Additionally, in this research, we collected the levels of CD3^+^ / CD4^+^ T_h_ cells in these patients with OLP and found that the T_h_ values of patients with OLP and positive *Candida* infection were 27–35 (T_h_ normal range as 27–51), with an average of 33.27, relatively lower than the rest average 37.40 within the normal value as 38–46. The observation deemed the immune level of patients with OLP and *Candida* was relatively weaker. Subsequently, the disorder of endocrine hormone level and reduction of host defense function might increase the vulnerability with *C. albicans*.

Studies revealed [19, 20] that hyphae growth and stability to maintain mycelial morphology were attributable to serum. Besides white plaque lesions, congestion, erosion, seepage and ulcer also exist among the lines of OLP lesions. Environmental conditions of these pathological states for *Candida* phase switching were all different. We preliminarily deem that this might be the reason why the *Candida* detection rates from erosive OLP were higher than non-erosive OLP, and such phenotypic variability of OLP might just be a competition between host's immune defense and stimulation from pathogen *C. albicans*.

### Biphasic expression of *HEGs* in *C. albicans* from OLP

#### Biphasic expression of *HWP1* and *HGC1*

To date, *HGC1* is acknowledged as the uniquely essential gene for hypha growth, which is an adjusting protein gene of cellular cycle G1 for mycelium morphology [21, 22]. In this study, the electrophoresis results of the RT-PCR products showed a full expression of *HWP1* and *HGC1* mRNAs in mycelial phase cells (Fig. 2a–f), and the real-time PCR measurement further presented trace expression of *HWP1* and *HGC1* mRNAs in yeast cells (Table 4 and Fig. 3a–h), indicating that *HWP1* and *HGC1* might be essential genes respectively for adhesion and morphologic function in pathogenicity of *C. albicans* in OLP. *HGC1* and *HWP1* confer respective function in hyphae morphogenesis and invasion into host epithelia cell to induce OLP.

#### Biphasic expression of *ALS3, ECE1*

*ALS3* is the gene which encodes the surface protein of cell wall. Its expression level is relatively high in cells, deeming its important significance in maintaining hyphae. It was once even acknowledged as the target gene in cellular immunity and antibody induction in *C. albicans*. In fact, *ALS3*-deficient mutants could normally adhere to early biofilm at the beginning of hypha formation, but the adhesion time is short, which makes cells fall off easily [23].

*ECE1* is proved to encode cell membrane protein. According to previous literature [24, 25], it is a polypeptide sequence composed of 271 amino acid residues and 34 amino acids, with no obvious correlation with the formation of hyphae, but formation of hyphea is incomplete in *ECE1*-deficient cell, with reduction of adhesion ability, demonstrating that *ECE1* might play an integral role in morphology maintenance and function improvement for hypha growth. However, later explorations tended to reveal that ECE1 correlated closely to the extension of hyphae, with an increasing expression in the process of mycelial grow [26]. Briefly, it has been inferred consensually that *ECE1* does not participate in morphogenesis of hypha formation.

Consistent to that *ALS3, ECE1* are not deemed to be the essential genes of *Candida* growth and the morphogenesis of hyphae production, the results in this research showed that *ALS3* and *ECE1* both expressed no obvious difference in yeast and mycelium phases (Fig. 2a–f), and might not be the essential genes for hyphae of *C. albicans* in OLP, while their roles in hyphae maintenance and adhesion could be indicated or further cared possibly.

#### Biphasic expression of *EFG1*

In fact, the morphologic phase switching of *C. albicans* is regulated by many signal pathways to ensure the genes *HEGs* to express. Among those signal pathways, *EFG1* is a star molecule currently. It is a feasible idea to control *C. albicans* through regulating *EFG1* to alter phase morphology [27], which might be potentially positive clues or basis for researches on susceptive drugs according to resistant genes.

*EFG1* plays a crucial role in multiple signaling pathways [28, 29] to regulate *HEGs* with other different signaling molecules. These pathways include endocytosis effect in the substrate: endocytosis effect in the substrate → Dck1 → Rac1^Efg1/Fol8^ → Czf1 → *HEGs*; Temperature/serum pathways: temperature → Hsp90 → serum → Ras1 → Cyr1 → cAMP → Tpk1/ Tpk2 → Efg1 → Flo8 → *HEGs*; the classic pH signaling pathways: pH → Rim21 → Rim8 → Rim13/20 → Efg1 → *HEGs*; N-acetyl glucosamine signaling pathways: N-acetyl glucosamine → Ngt1 → Efg1 → *HEGs*, and additionally the hormone levels, serum nitrogen concentration, methyl-methionine, and methionine etc.

In this research, *EFG1* mRNA expressions in yeast and mycelial phase cells showed no significant differences by the real-time PCR quantitative detection (Table 4 and Fig. 3a–h) indicating it was not *HEGs*. However, the experiments presented biofilms and hyphae forms were incomplete in *EFG1* expression inhibited cells, implicating that although *EFG1* mRNA had normal expression, it couldn't illustrate the specific expression of *EFG1*. Further experiments about qualitative and quantitative detection of EFG1 are meaningful.

## Conclusions

*C. albicans* and *Candida* prevalence in patients with OLP showed that isolation ratio from erosive group overweighed the non-erosive OLP patients within this data. *HWP1* was essential for adhesion and *HGC1* was essential for morphogenesis in pathogenicity of *C. albicans* in OLP. *HGC1* might be a unique essential gene for hypha morphogenesis, and *HWP1* was crucial in hyphae maintenance and adhesion. Besides, *ALS3, ECE1*, and *EFG1* played assistant roles in hyphae maintenance and adhesion. This experiment didn’t included other pathways such as Tup1, NRG1, Rfg1 [30] on the reverse biphasic state of expression. Follow-up researches could be launched to explore how to block the development of *Candida* pathogenesis in OLP.

## Data Availability

The datasets used and/or analyzed during the current study are available from the corresponding author on reasonable request.

## Abbreviations

*C. albicans*: *Candida albicans*
OLP: Oral lichen planus
HEGs: Hypha essential genes
*C. parapsilosis*: *Candida parapsilosis*
RT-PCR: Reverse transcriptase‐polymerase chain reaction
Real-time PCR: Real-time polymerase chain reaction
